# Prediction of Left Double-Lumen Tube Size by Measurement of Cricoid Cartilage Transverse Diameter by Ultrasound and CT Multi-Planar Reconstruction

**DOI:** 10.3389/fmed.2021.657612

**Published:** 2021-06-16

**Authors:** Chengchao Zhang, Xinlei Qin, Wenyi Zhou, Shuaijie He, Ao Liu, Yu Zhang, Zhigang Dai, Jiangwen Yin

**Affiliations:** Department of Anesthesiology, First Affiliated Hospital, School of Medicine, Shihezi University, Shihezi, China

**Keywords:** ultrasound, CT multi-planar reconstruction, cricoid cartilage transverse diameter, left double lumen tube, one lung ventilation

## Abstract

**Background:** Currently, there is no uniform standard for selecting the left double lumen tubes (LDLT). Advantages, such as safety and convenience of the ultrasonic technology, and measurement accuracy, make it more widely applied in the clinical anesthesia, and computed tomography (CT) multi-planar reconstruction (MPR) technology will certainly provide a more accurate measurement. For better application for thoracic surgery choice LDLT, relieving the injury to patients, and reducing the complications, this study will compare the two approaches.

**Methods:** The first part, 120 cases of patients were selected according to the height and gender; recording the patient's optimum LDLT and measurement the transverse diameter of the cricoid cartilage (TD-C) by ultrasound and CT MPR, and then obtained the TD-C range measurement by ultrasound and CT MPR corresponding to different types of LDLT. The second part, total of 102 patients were divided into the ultrasound group and the CT MPR group. In the ultrasound group, TD-C was measured by ultrasound, the corresponding size for intubation was selected based on the conclusions derived from the first part. In the CT MPR group, TD-C was measured by CT MPR, the corresponding size of LDLT based on the conclusions derived from the first part.

**Results:** In the first part, 120 patients were no significant difference in the basic characteristics (*P* > 0.05). The accuracy of selecting the LDLT by conventional experience, namely height and gender was 58.3%. Ultrasonic measurement TD-C range was as follows: 32 Fr <15.88, 35 Fr: 15.88–16.80, 37 Fr: 16.75–17.81, and 39 Fr > 17.80. CT MPR measurement TD-C range was as follows: 32 Fr <15.74, 35 Fr: 15.74–16.65, 37 Fr: 16.56–17.68, and 39 Fr > 17.65. In the second part, there was no significant difference in the basic characteristics between the two groups (*P* > 0.05). The accuracy of intubation in the ultrasound group was 90.2% and the corresponding in the CT MPR group was 94.1% (*P* > 0.05).

**Conclusions:** The accuracy of selecting the LDLT based on TD-C is significantly higher than conventional experience; it can significantly reduce the post-operative complications and there was no statistical significance in the accuracy of LDLT selected for TD-C measurement by ultrasound vs. CT, and both of them could be safely used for the evaluation before intubation under anesthesia in thoracic surgery.

## Introduction

Double-lumen endotracheal intubation and one-lung ventilation are often used to perform effective lung isolation in patients undergoing thoracic, mediastinal, cardiac, and vascular surgery (1). Left double lumen tubes (LDLT) are often used clinically as a pulmonary isolation device, with high safety and strong practicability (1, 2), which can be successfully applied to the right and left surgeries of most patients (3). The most commonly chosen sizes are 32 Fr, 35 Fr, 37 Fr, 39 Fr, and 41 Fr (4). However, for the choice of LDLT, there is still no uniform standard, and it is usually based on the experience of anesthetist, depending on the patient's gender and height (5), but the accuracy is poor, often leading to selection of a too large or too small LDLT (6). If the LDLT is too small, the LDLT's tip may be too deep and it may block the upper bronchial opening, and there may be greater airflow resistance to the trachea, or there may be tracheal compression injury caused by very small LDLT but excessive inflation of the cuff, or part of the trachea may not reach the carina, affected the visual field of the operation which can even leading to pulmonary isolation or separation failure. If the LDLT is too large, the thicker and harder tube may lead to bronchial or airway damage (4). Therefore, selection of an appropriate LDLT is effective and it significantly avoids the complications associated with an oversized or undersized LDLT.

Computed tomography (CT) has been proved to measure the diameter of the trachea and bronchus (7). A spiral CT scan of the chest with multi-planar reconstruction (MPR) yields cross-sectional, coronal, and sagittal images of the chest, and the angles of inclination can be adjusted to obtain a strict orthogonal section of the cricoid cartilage and left main bronchus (LMB), so that the inner and outer diameters and the anterior and posterior diameters of the trachea and left main bronchus are measured accurately (8, 9). All patients undergoing chest surgery are required to have a CT scan before surgery, so it could be included in clinical protocols of pre-operative radiological evaluation. The correlation between the diameters of the trachea and LMB based on CT scan, was determined a 0.75 coefficient for males and a 0.77 coefficient for females. And then one formula about the bronchial diameter and the tracheal diameter is: ID LBM (mm) = (0.45^*^ID trachea) + 3.3(mm) (10). With the popularity of ultrasound imaging, ultrasound can quickly gain access to the operating room, ICU, emergency rooms, and even under the bad environment of airway anatomy (11), and its simple, convenient, safe, real-time, and can be repeated measurement and has other advantages; thus, it is more widely applied in the pre-operative evaluation of patients requiring measurement of the airway and trachea diameter (11). To better apply to the selection of LDLT intubation in thoracic surgery during clinical anesthesia, reduce the patient's injury, and reduce complications, this study intends to explore the accuracy of the ultrasonic contrast CT MPR technique to measure the transverse diameter of the cricoid cartilage (TD-C) to guide the selection of the LDLT size.

## Materials and Methods

### Design

This study was divided into the following two parts: the first part was an observational study, and the second part was a prospective, double-blind, randomized controlled intervention study, which was approved by the Ethics Committee of the First Affiliated Hospital of Shihezi University (2019-096-01), and written informed consent was obtained from all of the subjects participating in the trial and the study was registered in The Chinese Clinical Trial Registry (ChiCTR1900025963). From August 2019 to August 2020, 232 patients were enrolled at the First Affiliated Hospital of Shihezi University School of Medicine for elective thoracic surgery, and 222 patients were eventually enrolled in this study.

### Sample

The participants were recruited by thoracic surgeons. The inclusion criteria were as follows: American Society of Anesthesiologists (ASA) Classes I-II, Cormack-Lehane views grade I-II, thoracic surgery under general anesthesia surgery, age from 18 to 80 years, pre-operative chest high-resolution CT examination within a month, LDLT was used during anesthesia, and the patient was informed about the study and he/she or his/her family signed the informed consent form. The exclusion criteria were as follows: predicted difficult intubation, difficulty in opening the mouth, small jaw deformity, ultrasonic detection of abnormal cartilage ring morphology, cricoid wall hyperplasia, attachment, tumor and shape change in the main airway, pre-operative hoarseness or sore throat, previous laryngeal or neck surgery, or diseases that cause shrinkage of the trachea (5).

The first part of the study was conducted from August 2019 to March 2020. A total of 124 patients were randomly enrolled, and 120 patients met the inclusion and exclusion criteria. In the second part of the study, 108 patients were enrolled from April 2020 to August 2020, and 102 patients were statistically analyzed.

According to the preliminary experimental results, we found that the accuracy rate of selecting the LDLT with conventional experience (height, gender) was about 60%. On examining the efficacy (α = 0.05, β = 0.1), the accuracy rate of selecting the LDLT when measuring the TD-C was 90%, which was considered to be statistically significant. The sample size was calculated to be at least 50 patients in each group.

### Randomization

Using computer-generated random numbers, continuous patients were randomly assigned to the ultrasound group or the CT MPR group in a 1:1 ratio. The researchers who measured the TD-C, the anesthesiologists who performed endotracheal intubation, and patients were unaware of the grouping.

**Figure 1:**
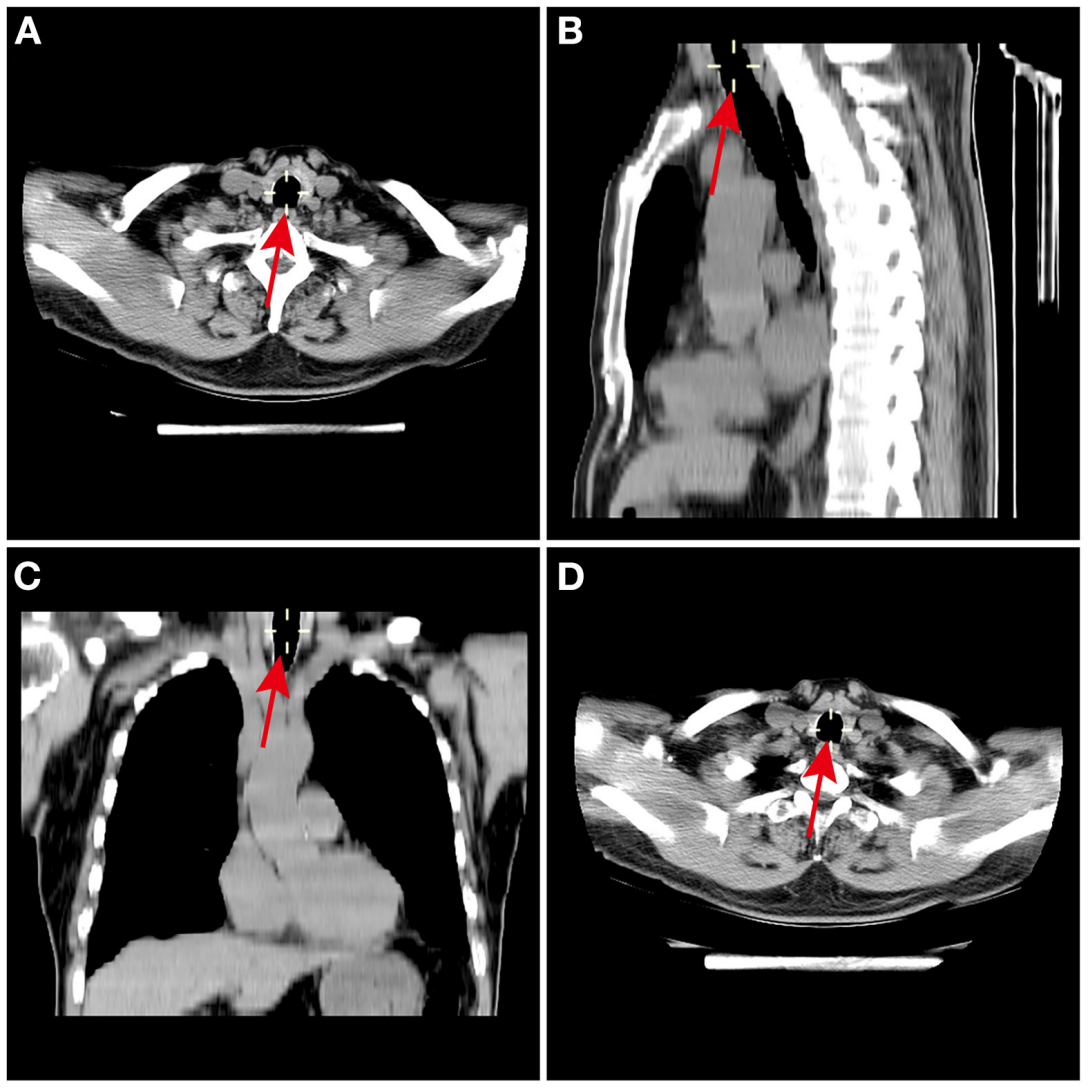
Measurement of the cricoid cartilage using chest HRCT scan MPR. The MPR of the cricoid cartilage was performed using the **(A)** axial, **(B)** sagittal, and **(C)** coronal slices. The declination of TD-C was corrected in 3 dimensions to obtain a strictly orthogonal cut of the cricoid cartilage axis. **(D)** The TD-C were measured on the MPR at the lower border of the cricoid ring. TD-C, transverse diameter of cricoid cartilage.

The radiologists, who were not aware of the grouping, and they used the CarestreamPACS software to reconstruct and measure the patient's trachea in MPR using CT. The tilt of the cartilage was adjusted to obtain a strict vertical section of the cartilage's subsurface (Figure 1). The cursor was used to measure the TD-C and its shape. To reduce the measurement error, the image was enlarged to 400% for measurement, and the average value was taken after obtaining repeated measurements three times.

**Figure 2:**
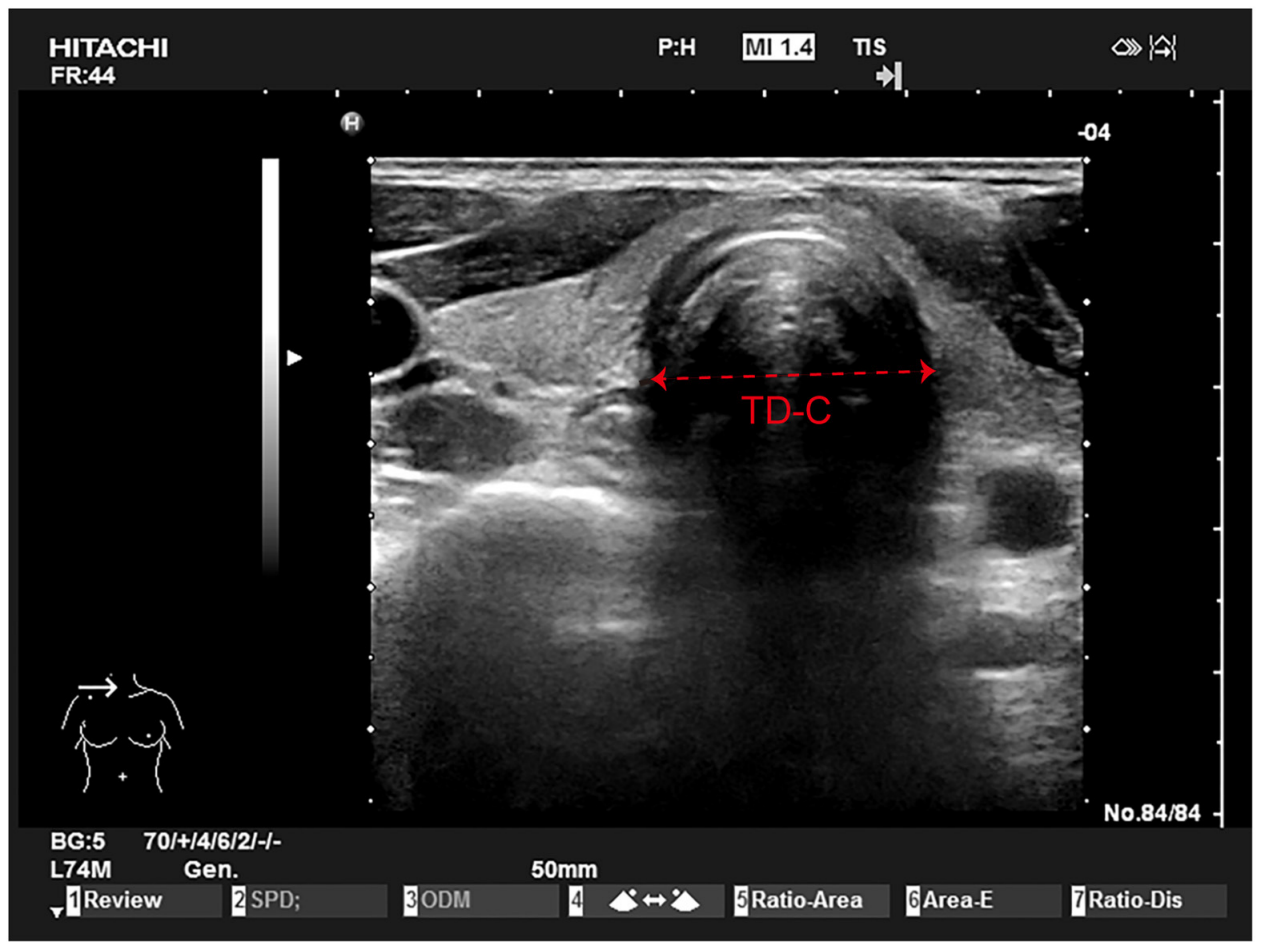
Tracheal ultrasound examination. A linear 5 to 10 MHz probe is placed perpendicularly to the neck just above the sternoclavicular junction in transverse section (left). The outer tracheal diameter is measured on this transverse view (mm).

Patients get into the operating room, the jaw was slightly tilted back, the anesthesiologist, who was trained by professional physician and not know the study plan, will probe ultrasonic probe with coupling agent after long axis with the neck on the sternoclavicular articulation point 0.5 cm place to get the cricoid cartilage parallel plane image, obtain patients expiratory pause at the end of the clearance of cricoid cartilage ring diameter image (Figure 2), the average value was taken after obtaining repeated measurements 3 times.

### Intervention

After entering the operating room, all of the patients were routinely monitored. Radial artery puncture was performed under local anesthesia, and invasive arterial pressure was monitored. After 3 min of pre-oxygenation (100% O_2_, 5 L/min), intravenous injection of sufentanil 0.5 μg/kg and propofol 1.5–2.5 mg/kg was performed for anesthesia induction. After the patient's consciousness disappeared, cisatracurium 0.2 mg/kg was injected intravenously, and positive pressure ventilation by a face mask was performed. Bronchial intubation was performed after positive pressure ventilation 4 min. The tidal volume was 6–8 ml/kg during one-lung ventilation, positive end-expiratory pressure (PEEP) was 4–6 cmH_2_O, and the respiratory rate was 12–20 times/min. Propofol, remifentanil, and cisatracurium were continuously pumped for anesthesia maintenance. While suturing the skin, the patients received self-controlled analgesia pump for post-operative pain management. At the end of the operation, the LDLT was retreated to the trachea and the patient was sent to the thoracic surgery ICU for mechanical ventilation and monitoring under anesthesia.

All of the bronchial intubations were performed by an anesthesiologist with at least 5 years of thoracic anesthesia experience. The anesthesiologist was not aware of the study method or grouping. First, select appropriate size of LDLT, the glottis was exposed with a video laryngoscope and the LDLT tip was inserted into the glottis under direct vision. advance the LDLT until a slight resistance is perceived, and the LDLT was continuously pushed to the expected depth. The expected depth (cm) of the LDLT = 12+ patient height (cm) /10. After adequate lubrication of the fiberoptic bronchoscope (FOB), introduce it into the tracheal lumen of the LDLT and identify the carina, and then verify the correct position of the bronchial lumen (the bronchial cuff must be barely visible). If the LDLT could not pass through the glottis due to severe resistance, the LDLT was continued to rotate anticlockwise 180° to continue the attempt to advance the LDLT. If the LDLT could not reach the trachea or bronchus due to severe resistance, intubation was performed with a smaller LDLT. After the intubation was complete, the anesthetist inserted the FOB to adjust the LDLT to the ideal location. Repeat the FOB control after patient position changes and throughout the intervention if necessary (12).

#### The First Part

**Figure 3:**
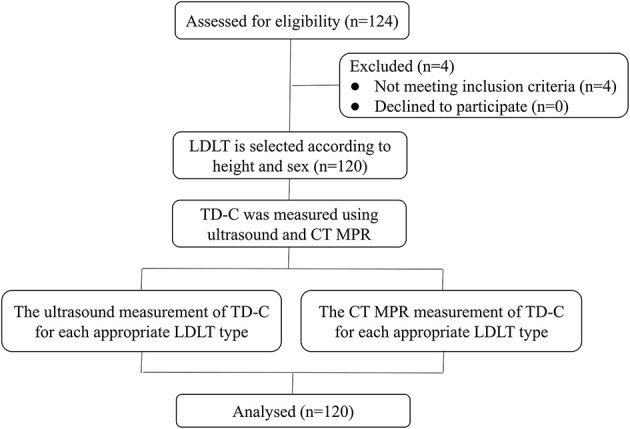
Study flowchart of patients in the first part. TD-C, transverse internal diameter of cricoid cartilage; LDLT, left double lumen tube; MPR, multi-planar reconstruction.

Anesthesiologists selected the LDLT according to the conventional experience, such as the patient's height and gender, and recorded the optimal LDLT size (recorded the LDLT size and judged the LDLT as too large, too small, or appropriate according to the judgment standard). If the LDLT was too large, it was recorded as the adjacent smaller LDLT size; If the LDLT was too small, it was recorded as the adjacent larger LDLT size. The corresponding TD-C by ultrasound and CT MPR of the patient were collected. The data were statistically analyzed to obtain the different sizes of LDLT corresponding to ultrasound and CT MPR measurement of the TD-C range (Figure 3).

#### The Second Part

In the ultrasound group, the TD-C was measured by ultrasound. The range of TD-C in the first part was applied to select the corresponding size for intubation. In the CT MPR group, TD-C was measured by CT MPR, the range of TD-C obtained in the first part was applied to select the corresponding size for intubation, and the accuracy rate of the LDLT selected in the two groups was compared.

## Observational Index

### Appropriate Standard for the LDLT

The main standard was that the LDLT was inserted smoothly (including the glottis, laryngeal, and bronchial areas) without resistance, the location of FOB inspection was correct and it arrived at the pre-determined bronchial level after adjustment, the intraoperative lung isolation was satisfactory, and the end-tidal carbon dioxide partial pressure (PetCO_2_) was maintained at 35–45 mmHg. Objective criteria were injected air into the cuff. When the pressure inside the LDLT was 25 mmHg, it was stopped and connected to the anesthesia machine, when the peak pressure of positive pressure ventilation airway was below 30 cm H_2_O, the air leakage phenomenon was adjusted (13).

### Oversized Standard for the LDLT

There was obvious resistance when the LDLT entered the trachea or bronchus, or the LDLT tip could not enter the bronchus after being guided by a fiberoptic bronchoscope. Good pulmonary isolation could be achieved by injecting <1 ml of air into the bronchial cuff and <2 ml of air into the main tracheal cuff (13).

### Undersized Standard for the LDLT

When the cuff pressure was adjusted to the standard value with the manometer after the LDLT was inserted successfully, the two lungs of the patients with airway leakage who needed injection of more than 3 or 6 ml of air into the two cuff could be satisfactorily isolated, and the LDLT size was considered to be too small (13).

### Pulmonary Isolation Effect

Satisfactory isolation: clear breathing sound during ventilation of the two lungs separately, airway resistance increased <10 cmH_2_O after blocking ventilation of one side; Unsatisfactory isolation: incomplete respiratory isolation after blocking one side, or significantly increased ventilation resistance > 10 cm H_2_O; No isolation: the two lungs could not be isolated at all, and no change was observed between blocking and not blocking.

### Subglottic Resistance

Zero: no resistance; 1: Slight resistance; 2: Moderate resistance; the trachea met significant resistance under the glottis, but it could pass through the glottis by rotating the LDLT; 3: Heavy resistance, even by rotating the LDLT, it could not pass under the glottis, it must be replaced with a smaller LDLT.

### Effects of Lung Collapse

Ten and 20 min after pleurotomy, a chest surgeon unaware of the grouping used a verbal rating scale (VRS) to evaluate the extent of lung collapse. Zero: Not collapsed at all; 10: Completely collapsed.

### Hoarseness at 24 and 48 h After Surgery

An uninformed physician assessed the patient's hoarseness. Zero: no hoarseness; 1: The patient was aware of it; 2: Onlookers can detect; 3: Loss of voice.

### Degree of Sore Throat at 24 and 48 h After Surgery

Zero: No sore throat; 1: Mild, sore throat only when swallowing; 2: Moderate, persistent sore throat, aggravated when swallowing; 3: Severe, throat pain affecting the patient's eating, and analgesic drugs were needed (11).

Other factors included age, gender, height, weight, Body Mass Index (BMI), the time required for intubation (it was defined as the time that the LDLT from the oral cavity to the expected depth), intubation times, LDLT changing conditions, Cormack-Lehane views grade I-II, the pressure and air in the trachea and bronchial cuff.

### Statistical Analysis

SPSS 22.0 was used for statistical analysis of the data. The Kolmogorov-Smirnov test is used to check whether the data fits normal distribution, Continuous variables conforming to a normal distribution were expressed as mean ± standard deviation, continuous variables not conforming to normal distribution were expressed as median and inter-quartile range, counting data were expressed as number and percentage, and the independent-samples *t*-test was used for inter-group analysis measurement date such as age, height, weight, TD-C between different genders. The χ^2^ test was used to compare the enumeration data between the sexes and the intubation accuracy of the two groups. The classified variables were compared using Fisher exact test. Pulmonary isolation effect and subglottic resistance were analyzed using a non-parametric test because the variables did not satisfy the criteria for normality. In all of the statistical analyses, *P* < 0.05 was considered to be statistically significant.

## Results

**Figure 4:**
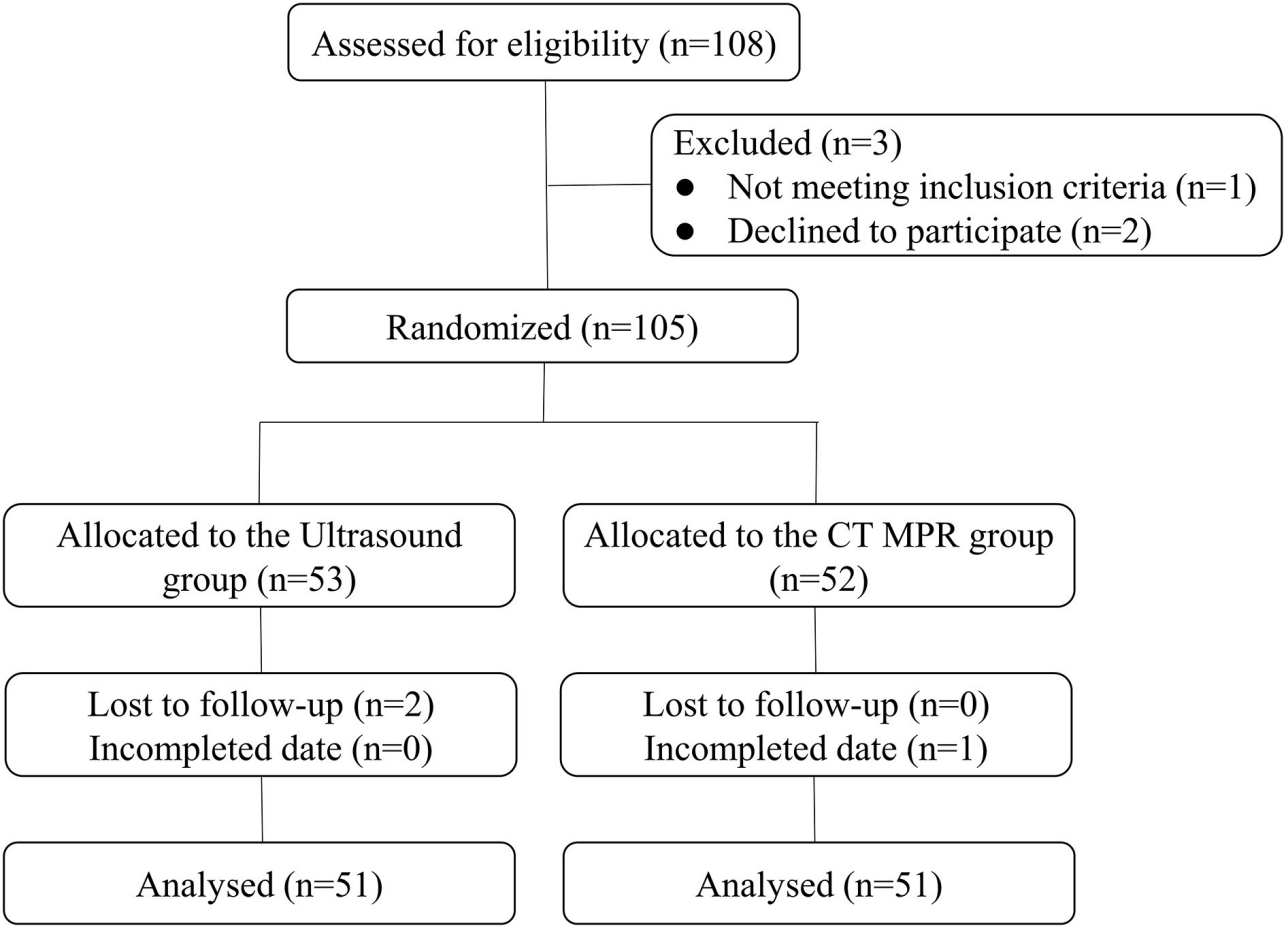
Study flowchart of patients in the second part.

In the first part, a total of 124 patients were assessed for eligibility. After exclusions (four patients did not meet the inclusion criteria), 120 patients were enrolled in this study (Figure 3). In the second part, among the 108 eligible patients, after exclusions, 102 patients were enrolled: 51 were randomized to the ultrasound group and 51 to the CT MPR group (Figure 4).

### In the First Part

A total of 120 patients met the inclusion and exclusion criteria, and finally statistically analyzed. There were no statistically significant differences in age, BMI, operation time, surgical site, or ASA classification between the two groups (*P* > 0.05). Compared with female patients, height, weight, and TD-C values were significantly increased in male patients (*P* < 0.05). The intubation time and intubation times of female patients were significantly higher than those of male patients (*P* < 0.05). In male patients, the shape of the cricoid cartilage was round (87.2%); while in female patients, the shape of the cricoid cartilage was oval (81.0%) (*P* < 0.05). Male patients had the maximum 37 Fr selection (62.8%), followed by 39 Fr selection (33.3%); and female patients had the maximum 37 Fr selection (45.2%), followed by 35 Fr selection (42.9%) (Table 1).

**Table 1:** Comparison of general conditions of male and female patients.

	**Male (*n* = 78)**	**Female (*n* = 42)**	***P*-value**
Age (y)	54.2 ± 15.7	52.4 ± 10.4	0.631
Height (cm)	171.4 ± 6.2	160.5 ± 4.8	<0.001
Weight (kg)	72.0 ± 14.5	65.0 ± 7.6	0.011
Intubation time (s)	35.3 ± 11.0	46.4 ± 13.2	<0.001
Operating time (h)	3.3 ± 1.5	3.8 ± 1.4	0.128
BMI (kg/m^2^)	24.6 ± 4.4	25.1 ± 2.6	0.577
Ultrasound TD-C	17.62 ± 0.63	16.71 ± 0.63	0.000
CT MPR TD-C	17.42 ± 0.58	16.59 ± 0.62	0.000
Operating site			0.242
Right (*n* = 86)	55 (70.5%)	31 (73.8%)	
Left (*n* = 34)	23 (29.5%)	11 (26.2%)	
ASA classify			0.652
I (*n* = 86)	56 (71.8%)	30 (71.4%)	
II (*n* = 34)	22 (28.2%)	12 (28.6%)	
Appropriate LDLT			<0.001
39Fr (*n* = 29)	26 (33.3%)	3 (7.1%)	
37Fr (*n* = 68)	49 (62.8%)	19 (45.2%)	
35Fr (*n* = 21)	3 (3.9%)	18 (42.9%)	
SLT 7.0 (*n* = 2)	0 (0.0%)	2 (4.8%)	
Intubation times			0.003
1 (*n* = 108)	72 (92.3%)	36 (85.7%)	
2 (*n* = 12)	6 (7.7%)	6 (14.3%)	
The shape of TD-C			0.02
Round (*n* = 76)	68 (87.2%)	8 (19.0%)	
Oval (*n* = 44)	10 (12.8%)	34 (81.0%)	

*Date are mean ± standard deviation or number (percentage). BMI, body mass index; TD-C, transverse internal diameter of cricoid cartilage; ASA, American Society of Anesthesiologist; LDLT, left double lumen tube; SLT, single lumen tube*.

Ultrasonic measurement TD-C range: 32 Fr <15.88; 35 Fr: 15.88 ~ 16.80; 37 Fr: 16.75 ~ 17.81; 39 Fr > 17.80. CT MPR measurement TD-C range: 32 Fr <15.74; 35 Fr: 15.74 ~ 16.65; 37 Fr: 16.56 ~ 17.68; 39 Fr > 17.65 (Table 2).

**Table 2:** The range of appropriate LDLT.

**Appropriate LDLT**	**Ultrasound TD-C**	**CT MPR TD-C**
32Fr	<15.88	<15.74
35Fr	15.88~16.80	15.74~16.65
37Fr	16.75~17.81	16.56~17.68
39Fr	17.80~18.88	17.65~18.52

*Values are expressed as 95% confidence interval [CI].MPR, multi-planar reconstruction*.

Among the 120 patients, the LDLT size was too large in 22 patients (18.3%), suitable in 70 patients (58.3%), and too small in 28 patients (23.4%). The large group had the longest intubation time (48.4 ± 9.2 s), and 10 patients (45.4%) had slight resistance when intubation passed through the subglottic area; 48 patients (68.6%) in the appropriate group had slight resistance when intubation passed; and 22 patients (78.6%) in the subglottic group had no resistance. The effects of lung isolation, pulmonary collapse, hoarseness, and sore throat 24 and 48 h after surgery in the appropriate group were significantly better than those in the oversized and undersized groups (*P* < 0.05) (Table 3).

**Table 3:** Comparison of observation indexes among the three groups.

	**Oversize**	**Appropriate**	**Undersize**	***P*-value**
	**(*n* = 22)**	**(*n* = 70)**	**(*n* = 28)**	
Intubation time (s)	48.4 ± 9.2	32.2 ± 8.5	36.8 ± 11.2	<0.001
Intubation times				0.002
1	15 (68.2%)	70 (100.0%)	23 (82.1%)	
2	7 (31.8%)	0 (0.0%)	5 (17.9%)	
Subglottic resistance				0.004
0	1 (4.5%)	19 (27.1%)	22 (78.6%)	
1	10 (45.4%)	48 (68.6%)	6 (21.4%)	
2	6 (27.3%)	3 (4.3%)	0 (0.0%)	
3	5 (22.8%)	0 (0.0%)	0 (0.0%)	
Pulmonary isolation				0.009
Satisfactory isolation	10 (45.5%)	49 (70.0%)	6 (21.4%)	
Unsatisfactory isolation	9 (41.0%)	21 (30.0%)	21 (75.0%)	
No isolation	3 (13.5%)	0 (0.0%)	1 (3.6%)	
Effects of lung collapse				<0.001
No collapse	3 (13.6%)	0 (0.0%)	2 (7.1%)	
Mild collapse	2 (9.1%)	0 (0.0%)	7 (25.0%)	
Severe collapse	13 (59.1%)	44 (62.9%)	13 (46.4%)	
Completely collapse	4 (18.2%)	36 (37.1%)	6 (21.5%)	
Hoarseness at 24 h				<0.001
0	6 (27.3%)	37 (52.9%)	16 (57.1%)	
1	10 (45.4%)	33 (47.1%)	11 (39.3%)	
2	6 (27.3%)	0 (0.0%)	1 (3.6%)	
Hoarseness of 48 h				<0.001
0	11 (50.0%)	64 (91.4%)	22 (78.6%)	
1	11 (50.0%)	6 (8.6%)	6 (21.4%)	
2	0 (0.0%)	0 (0.0%)	0 (0.0%)	
Sore throat at 24 h				<0.001
0	0 (0.0%)	19 (27.1%)	4 (14.3%)	
1	10 (45.5%)	46 (65.8%)	16 (57.1%)	
2	12 (54.5%)	5 (7.1%)	8 (28.6%)	
Sore throat at 48 h				<0.001
0	7 (31.8%)	53 (75.7%)	16 (57.1%)	
1	14 (63.6%)	17 (24.3%)	11 (39.3%)	
2	1 (4.6%)	0 (0.0%)	1 (3.6%)	

*Date are mean ± standard deviation or number (percentage). Subglottic resistance: 0, no resistance; 1, slight resistance; 2, moderate resistance; 3, heavy resistance. Hoarseness: 0, no hoarseness; 1, the patient is aware of it; 2, onlookers can detect*.

### In the Second Part

A total of 102 patients were included and randomly divided into the ultrasound group and the CT MPR group. There were no statistically significant differences in gender, age, height, weight, BMI, ASA classification, surgical site, duration of operation, and number of intubations between the two groups (*P* > 0.05), and the number of intubations in the two groups was once (Table 4).

**Table 4:** Comparison of general conditions of two groups.

	**Ultrasound (*n* = 51)**	**CT MPR (*n* = 51)**	***P-*value**
Age (y)	53.3 ± 13.3	57.7 ± 15.3	0.124
Gender			0.836
Female	18 (35.3%)	17 (33.3%)	
Male	33 (64.7%)	34 (66.7%)	
Weight (kg)	68.7 ± 9.3	66.2 ± 10.4	0.214
Height (cm)	166.5 ± 8.9	165.5 ± 7.9	0.418
BMI (kg/m^2^)	25.2 ± 1.4	24.8 ± 1.4	0.171
ASA classify			0.562
I	41 (80.4%)	38 (74.5%)	
II	10 (19.6%)	13 (25.5%)	
Operating site			0.724
Right	35 (68.6%)	33 (64.7%)	
Left	15 (31.4%)	18 (35.3%)	
Operating time (h)	3.5 ± 1.5	3.6 ± 1.7	0.154
Intubation time (s)	28.2 ± 7.4	26.6 ± 7.0	0.752
Intubation times
1	51 (100.0%)	51 (100.0%)	
2	0 (0.0%)	0 (0.0%)	

*Date are mean ± standard deviation or number (percentage)*.

In the ultrasound group, two cases were too large and three cases were too small with respect to choosing the LDLT, and the accuracy rate of choosing an appropriate LDLT was 90.2%. In the CT MPR group, 2 cases were too large and 1 case was too small, and the intubation accuracy was 94.1%. There was no statistically significant difference in the tube selection accuracy between the two groups (*P* > 0.05) (Table 5).

**Table 5:** Comparison of LDLT accuracy between the two groups.

	**Ultrasound (*n* = 51)**	**CT MPR (*n* = 51)**	***P-*value**
Oversized	2	2	0.128
Appropriate	46	48	0.846
Undersized	3	1	0.215
Accuracy rate	90.20%	94.10%	0.097

*Date are number (percentage)*.

In the two groups of patients, choosing the number of each size of LDLT, TD-C value, the trachea and bronchus cuff volume, subglottic resistance, lung isolation effect, lung collapse effect, and post-operative 24 and 48 h hoarseness and sore throat showed no statistical difference (*P* > 0.05), and some patients (22 vs. 24) at 24 h after surgery had a mild sore throat, and only a few patients (3 vs. 5) at 24 h after surgery had mild hoarseness (Table 6).

**Table 6:** Comparison of observation indexes among the two groups.

	**Ultrasound (*n* = 51)**	**CT MPR (*n* = 51)**	***P-*value**
Appropriate LDLT			0.421
39Fr	14 (27.5%)	10 (19.6%)	
37Fr	24 (47.1%)	30 (58.8%)	
35Fr	12 (23.5%)	10 (19.6%)	
SLT7.0	1 (1.9%)	1 (2.0%)	
TD-C (mm)	17.45 ± 0.78	17.25 ± 0.71	0.182
Trachea cuff volume (ml)	4.12 ± 0.56	4.00 ± 0.53	0.275
Bronchial cuff volume (ml)	2.06 ± 0.31	2.00 ± 0.35	0.369
Subglottic resistance			0.687
0	45 (88.2%)	43 (84.3%)	
1	6 (11.8%)	8 (15.7%)	
2	0 (0.0%)	0 (0.0%)	
3	0 (0.0%)	0 (0.0%)	
Pulmonary isolation			0.246
Satisfactory isolation	46 (90.2%)	44 (86.3%)	
Unsatisfactory isolation	5 (9.8%)	7 (13.7%)	
No isolation	0 (0.0%)	0 (0.0%)	
Effects of lung collapse			0.091
No collapse	45 (88.2%)	47 (92.2%)	
Mild collapse	6 (11.8%)	4 (7.8%)	
Severe collapse	0 (0.0%)	0 (0.0%)	
Completely collapse	0 (0.0%)	0 (0.0%)	
Hoarseness at 24 h			0.239
0	48 (94.1%)	46 (90.2%)	
1	3 (5.9%)	5 (9.8%)	
2	0 (0.0%)	0 (0.0%)	
Hoarseness of 48 h			0.585
0	50 (98.0%)	49 (96.1%)	
1	1 (2.0%)	2 (3.9%)	
2	0 (0.0%)	0 (0.0%)	
Sore throat at 24 h			0.512
0	26 (51.0%)	22 (43.1%)	
1	22 (43.1%)	24 (47.1%)	
2	3 (5.9%)	5 (9.8%)	
Sore throat at 48 h			0.266
0	45 (88.2%)	42 (82.4%)	
1	5 (9.8%)	7 (13.7%)	
2	1 (2.0%)	2 (3.9%)	

*Date are mean ± standard deviation or number (percentage)*.

In the first part, 28 (23.4%) patients' LDLT was too small, 22 (18.3%) patients' LDLT was too large, and 70 (58.3%) patients' LDLT was appropriate. In the second part, according to the TD-C selection, the LDLT size was too large in four patients (3.9%), too small in four patients (3.9%), and suitable in 94 patients (92.2%), with a statistically significant difference (*P* < 0.05) (Table 7).

**Table 7:** Comparison of accuracy between the first part and the second part.

		**The first part (*****n*** **=** **120)**			**The second part (*****n*** **=** **102)**			***P-*value**
		**Undersize**	**Appropriate**	**Oversized**	**Undersize**	**Appropriate**	**Oversized**	
Male	32Fr	0	0	0	0	1	0	
	35Fr	1	1	0	1	6	0	
	37Fr	11	37	6	2	39	1	
	39Fr	0	13	9	0	20	0	
	All	12 (15.4%)	51 (65.4%)	15 (19.2%)	3 (4.3%)	66 (94.3%)	1 (1.4%)	<0.001
Female	32Fr	0	0	0	0	1	0	
	35Fr	12	11	2	1	9	1	
	37Fr	4	8	5	0	15	2	
	39Fr	0	0	0	0	3	0	
	All	16 (38.1%)	19 (45.2%)	7 (16.7%)	1 (3.1%)	28 (87.5%)	3 (9.4%)	<0.001
All		28 (23.4%)	70 (58.3%)	22 (18.3%)	4 (3.9%)	94 (92.2%)	4 (3.9%)	0.001

*Date are number (percentage)*.

## Discussion

We found that in the first part of this study, by using ultrasonic and CT MPR, we could obtain the range of TD-C, then the second part was used to verify this method, as a result the success rate of intubation was more than 90%. Too large or too small of LDLT can increase the intubation time and the number of intubations, subglottic resistance, post-operative sore throat and hoarseness, can lead to lung failure isolation and one-lung collapse is not complete. The longer intubation duration and the more times of intubations in female patients than in male patients, we analyzed is due to the more constricted airway. In female, subglottic resistance is often encountered during intubation. The cricoid cartilage of female is oval, while in male, it is mostly round. In our study, the elliptic shape of the cricoid cartilage often leads to a larger choice of LDLT size, intraoperative lung isolation is not satisfactory, and post-operative complications, sore throat, and hoarseness. This is also where we need to pay attention in the future to continue our experiments with LDLT size selection.

At present, the LDLT is usually selected according to the conventional experience, such as gender, height, when the anesthesia department of most domestic hospitals conducts thoracic surgery, but it has low accuracy. Since there is no significant correlation between patient height and airway diameter, this approach tends to lead to inappropriate LDLT selection in Asians (14). According to Miller's anesthesiology, in female with height <152 cm, 32 Fr should be chosen, height <160 cm, 35 Fr should be chosen, and height > 160 cm, 37 Fr should be chosen. For male with height <160 cm, the 37 Fr LDLT should be selected; with height <170 cm, 39 Fr LDLT should be selected; with height > 170 cm, 41 Fr LDLT should be selected (15). The advantages of this method are as follows: it is simple and easy to use. The disadvantages of this method are as follows: the predicted by this method is the median value, and the LDLT size selected was not suitable for all patients due to the great individual variation of the airway size in patients with the same height range. Therefore, the specificity of this method is poor and the positive predictive value is low. This selection method is based on Europeans and Americans. Since Asians are generally smaller than Europeans and Americans, this method may not be applicable to Asians, especially Asian female.

It is generally believed that the correct LDLT size should be determined according to the LMB (16). However, LDLT selected according to the LMB often encounters significant subglottic resistance, especially in female patients (17). Since the cricoid cartilage is the narrowest part of the trachea, its diameter may be a determinant of the appropriate LDLT size (18). Since the shape of the LMB is not circular or elliptical, and it is not perpendicular to the cross section in space, it is difficult to measure it (12). Parab pointed out that the cricoid cartilage is almost always oval in shape and that in 75% of cases (19), the anterior and posterior diameter is longer. Kim D describes the circular shape of the lower margin of the cricoid cartilage (16). This difference may be caused by differences in race and method, and further research is needed to elucidate the factors influencing its structure. Similar to the LMB, the TD-C cannot be accurately predicted due to the poor correlation between height and the TD-C (17).

In recent years, the application of ultrasound technology in clinical anesthesia has developed rapidly, and it has become one of the hot spots in clinical research (20). CT MPR technology has been proved to be able to accurately measure the diameter of the trachea and bronchus. Ultrasound and CT MPR have respective advantages in tracheal measurement and airway assessment (16), but few scholars at home and abroad have compared the two methods to evaluate the accuracy of the LDLT size. Kayashima K proved that ultrasonic measurement of the tracheal diameter combined with the patient height and gender could accurately guide the selection of the LDLT size (17). Nain and others experimented measuring the LMB by CT and the correlation between ultrasonic measurement of the tracheal diameter, the result is not encouraging, this means that the diameter of the LMB cannot be predicted by measuring the diameter of the trachea (21). Gu experimented measuring the trachea diameter by the neck ultrasound and CT; both with strong correlation, can better reflect the real situation of the trachea, but the disadvantage is not for the two kinds of measurement methods were compared (22). Although this experiment proves the LDLT in TD-C measurement accuracy, ultrasound and CT MPR show no difference, but CT MPR and its advantages, we can see the anteroposterior diameter of cricoid cartilage, when the patients' cricoid cartilage for the oval shape is irregular, we can even combine the transverse diameter and anteroposterior diameter to predict the size of LDLT.

The CT MPR technique can provide more accurate information needed for intubation. Chest CT can provide the following information: (1) the values of transverse diameters of the trachea and bronchus, their patency, changes in the inner diameter, stenosis, compression, distortion, and angulation; (2) whether the carina is shifted to the left or right, whether the plane of the left and right bronchi is consistent with the coronal plane, and how it is shifted; (3) the position of the bronchial opening of the upper lobe and its distance from the carina; (4) can clearly observe the bilateral lung structure and pulmonary vessels, especially the ventilatory side of the lung (23). Based on the advantages of CT, we found that the included angle between the trachea and the LMB was significantly different among different patients. Among them, patients with large angle formation had longer intubation time, increased intubation times, and were more likely to have sore throat and hoarseness on the first day after surgery.

Difficult airway management can be challenging for anesthesiologists, especially in thoracic anesthesia, the incidence of difficult airway was higher than that of the general patients, and the video laryngoscopy was successfully used in difficult intubation patients (24). Patients with normal size and shape of trachea and bronchial were enrolled in this study, however, in patients with difficult airways, measuring airway diameter alone is not sufficient, the LDLT with an embedded camera can confirm the LDLT's position and minimize the requirement for a bronchoscope and avoid the need to open the airway. Even when using tubes with embedded cameras, bronchoscopes can still occasionally be necessary (25).

Because LDLT has a large outside diameter and a pre-shaped tip, it can easily cause airway damage once the LDLT is not selected properly; thus, hoarseness and sore throat are common complications after bronchial intubation (26–28). The first part of this experiment with the LDLT size showed the probability of an oversized group of severe post-operative sore throat than the appropriate group, 12 cases of patients with severe post-operative sore throat suffered from serious resistance and the intubation time was more than 50 s, and eight cases of patients experienced more than two episodes of intubation; and in the second part, all of the patients did not develop severe post-operative sore throat.

There are still some deficiencies and improvements needed in this experiment. First, the TD-C was measured by ultrasound and CT MPR in this experiment, while the LMB and its shape were not measured. The accuracy of LDLT selection was analyzed by comparing the TD-C and LMB in subsequent experiments. Second, all of the patients in this experiment came from the same region and visited the same hospital; thus, the sample representation was general. Third, in the second part of the experiment, two patients in the ultrasound group had difficulty in intubation, resulting in insufficient ventilation. CT images showed that the angle between the trachea and LMB was relatively large, and subsequent experiments would consider the image of the trachea and bronchus angulation for LDLT.

## Conclusions

In summary, the conclusion of this experiment was that the TD-C range of ultrasonic measurement was as follows: 32 Fr <15.88, 35 Fr: 15.88–16.80, 37 Fr: 16.75–17.81, and 39 Fr > 17.80. The CT MPR measurement TD-C range was as follows: 32 Fr <15.74, 35 Fr: 15.74–16.65, 37 Fr: 16.56–17.68, and 39 Fr > 17.65. The accuracy rate of LDLT intubation in the ultrasound group was 90.2%, and that of LDLT intubation in the CT MPR group was 94.1%, with no significant difference in the intubation accuracy between the two groups.

## Data Availability Statement

The raw data supporting the conclusions of this article will be made available by the authors, without undue reservation.

## Ethics Statement

The studies involving human participants were reviewed and approved by the Ethics Committee of First Affiliated Hospital of Shihezi University (2019-096-01), and written informed consent was obtained from all of the subjects participating in the trial and the study was registered in The Chinese Clinical Trial Registry (ChiCTR1900025963). The patients/participants provided their written informed consent to participate in this study.

## Author Contributions

JY, ZD, and CZ contributed to the study conception and design. Material preparation, data collection and analysis were performed by CZ, XQ, WZ, and AL. SH and YZ analyzed the data. The first draft of the manuscript was written by CZ and XQ. All authors read and approved the final manuscript.

## Conflict of Interest

The authors declare that the research was conducted in the absence of any commercial or financial relationships that could be construed as a potential conflict of interest.
